# Nanomaterials as Ultrasound Theragnostic Tools for Heart Disease Treatment/Diagnosis

**DOI:** 10.3390/ijms23031683

**Published:** 2022-01-31

**Authors:** Edouard Alphandéry

**Affiliations:** 1 Sorbonne Université, Muséum National d’Histoire Naturelle, UMR CNRS 7590, IRD, Institut de Minéralogie, de Physique des Matériaux et de Cosmochimie, IMPMC, 75005 Paris, France; edouardalphandery@hotmail.com; 2 Nanobacterie SARL, 36 boulevard Flandrin, 75116 Paris, France; 3 Institute of Anatomy, UZH University of Zurich, Institute of Anatomy, Winterthurerstrasse 190, CH-8057 Zurich, Switzerland

**Keywords:** nanomaterials, nanotechnology, nanomedicine, nano-oncology, cancer, ultrasounds, high intensity ultrasounds, contrast agent, sonodynamic therapy

## Abstract

A variety of different nanomaterials (NMs) such as microbubbles (MBs), nanobubbles (NBs), nanodroplets (NDs), and silica hollow meso-structures have been tested as ultrasound contrast agents for the detection of heart diseases. The inner part of these NMs is made gaseous to yield an ultrasound contrast, which arises from the difference in acoustic impedance between the interior and exterior of such a structure. Furthermore, to specifically achieve a contrast in the diseased heart region (DHR), NMs can be designed to target this region in essentially three different ways (i.e., passively when NMs are small enough to diffuse through the holes of the vessels supplying the DHR, actively by being associated with a ligand that recognizes a receptor of the DHR, or magnetically by applying a magnetic field orientated in the direction of the DHR on a NM responding to such stimulus). The localization and resolution of ultrasound imaging can be further improved by applying ultrasounds in the DHR, by increasing the ultrasound frequency, or by using harmonic, sub-harmonic, or super-resolution imaging. Local imaging can be achieved with other non-gaseous NMs of metallic composition (i.e., essentially made of Au) by using photoacoustic imaging, thus widening the range of NMs usable for cardiac applications. These contrast agents may also have a therapeutic efficacy by carrying/activating/releasing a heart disease drug, by triggering ultrasound targeted microbubble destruction or enhanced cavitation in the DHR, for example, resulting in thrombolysis or helping to prevent heart transplant rejection.

## 1. Introduction

Ultrasound is commonly used to obtain an image of an internal organ or to examine a pregnant woman. It displays certain advantageous properties such as a relatively modest cost, an absence of toxicity due to the use of non-ionizing radiations and wide availability [1]. Certain improvements can be achieved by using contrast agents such as microbubbles (MB), allowing an increase in ultrasound resolution and a visualization of certain tiny parts of the organism such as the interior of blood vessels [2]. In addition, it has recently been suggested to use these contrast agents not only for diagnosis, but also for therapy, making these materials theragnostic ultrasound contrast agents (CA), [3]. Among the different uses of such compounds, the treatments of tumors and heart diseases are the most frequently described [3]. Although these two pathologies are very different from each other, their treatments may require contrast agents sharing some common general properties characterized by their ability to locally target a diseased region, detect abnormalities at cellular or sub-cellular level, and trigger a therapeutic activity locally to improve the benefit/risk ratio of the treatments of these two diseases.

**Figure 1:**
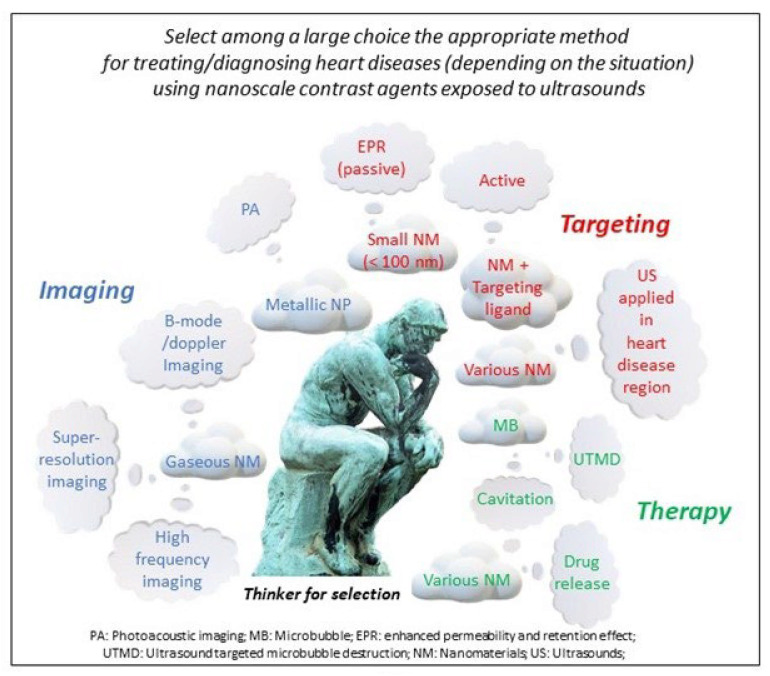
A schematic figure illustrating the large choice of methods used to treat or diagnose a cardiac disease by using a combination of contrast agents and ultrasounds. While gaseous nanomaterials can be used for ultrasound imaging, metallic ones can serve in photo-acoustic imaging. Targeting of the DHR to specifically image/treat this region can be achieved by designing NMs that target the DHR through passive, active, or magnetic targeting, and/or by applying ultrasound in this region. Therapeutic activity can be obtained through the release/activation of heart disease drugs under controlled conditions of ultrasound application and/or via ultrasound targeted microbubble destruction or cavitation, which can be enhanced in the presence of the contrast agent.

In this review, the different types of nanomaterials that can be used for the treatment and detection of heart disease, are first presented. They include microbubbles, nanobubbles, nanodroplets, biodegradable polymeric nano-capsules, hollow nanometric silica structures (HNSS), and magnetic nano-structures embedded within the pores of silica meso-structures. Second, the added value of ultrasound contrast agents in ultrasound imaging is presented. The resolution of ultrasonography, which is commonly used to detect various dysfunctioning heart parts, can be improved by using an ultrasonic wave for the excitation and detection of MB. On the one hand, such improved resolution can come from a refinement of the types of ultrasound beam that is employed, e.g., by using harmonic/sub-harmonic imaging or by increasing the frequency of the applied ultrasound or the speed of detection of the ultrasound beam. On the other hand, the contrast can be enhanced in the presence of MB, whose gas content can locally create a difference in acoustic impedance between the interior of MB and the surrounding heart tissue [4]. To ensure that the contrast occurs in the desired region of the diseased heart, a ligand can be attached to the MB, which especially recognizes such regions. To extend to types of ultrasonic contrast agent other than MB, photoacoustic imaging (PA) can be used. In this case, an exciting laser wave produces a thermoelastic expansion of a plasmonic NM, resulting in the emission of an ultrasonic wave that is then detected by ultrasonic imaging [5]. In PA, the contrast will essentially occur from the plasmonic effect at the NM surface, hence enabling PA to be used in combination with nanomaterials displaying surface plasmon wave effects such as those surrounded by Au materials. Third, I discuss the various ways in which CA can target a diseased heart region (DHR). When Cas display nanometric sizes, they can passively diffuse by the Enhanced permeability and retention effect (EPR) toward the DHR, a mechanism that can be enhanced under US application in a relatively similar manner as for tumor targeting via EPR [6]. Most interestingly, when the contrast is expected to arise from MBs whose sizes are larger than 1 μm, nanometric NDs passively diffuse by the Enhanced Permeability and Retention Effect (EPR) toward the DHR and once there, ND can be transformed into MB through acoustic droplet vaporization (ADV) [7]. Active targeting may also be employed. In this case, ligands are attached to CA, hence enabling CA to specifically target DHR covered by receptors binding to these ligands [8]. Fourth, CA exposed to ultrasound can have various therapeutic function. They may release drugs under controlled conditions and yield the destruction of thrombi followed by arterial recanalization [9]. Such a mechanism can be accompanied by the so-called ultrasound targeted microbubble destruction (UTMB), which can further improve drug delivery efficacy or support such mechanisms [10]. The different types of CA, their operating conditions as well as their applications for therapeutic and imaging applications for heart diseases are summarized in Table 1. Figure 1 presents the wealth of the different combinations of nanoscale contrast agents and ultrasounds which can be used to treat, image and/or target a diseased heart region..

## 2. Various Types of Nanomaterials Used as Theragnostic Ultrasound Contrast Agents for the Treatment/Diagnosis of Heart Diseases:

The properties of the different types of nanomaterials, which have been described as suitable ultrasound theragnostic contrast agents for the treatment or diagnosis of heart diseases, are presented in Table 1 and described below.

Microbubbles, which are spheroidal vesicles, are the most widely described contrast agents for use in imaging and treatment of cardiac diseases. While the MB coating material can be made of lipids or polymers or denatured proteins [40], those used for heart diseases mainly consist of an outer lipid layer consisting, for example, of macrogol 4000, DSPC, and palmitic acid for Sonovue [44], DSPC, DSPE-PEG2000 with/without DC-CHOL for cationic microbubbles (CMB) [45], or hydrated double-lipid-layers for nanoliposomes [26]. MB encloses an internal core filled with gases such as SF_6_ for Sonovue [46], or C_3_F_8_ for cationic MBs [45], to the high compressibility and ultrasound-responsive property of microbubbles [47] or an active principle. The average size of these materials is often reported to exceed 1 µm; microbubbles with a typical diameter of 1–8 μm [48] allow them to act as ultrasonic contrast agents, raising the question of whether they should be categorized as nanomaterials. In fact, several aspects argue in favor of such a categorization. First, MBs display a size distribution, which is often very large and include MBs of sizes below 100 nm, as is the case for Sonovue or for nanoliposomes (Table 1). Second, their size depends on the method used to measure it, leading to a difference by a factor of 2 between the average diameter of 1.6 µm measured by electro-impedance volumetric zone sensing and that of 0.8 µm estimated by laser diffraction [49]. Third, some MBs are mixed structures containing nanomaterials of nanometric size where the latter can be used to promote the association of an active principle with MBs [50]. Fourth, some MBs are derived from nanomaterial structures, especially when they are created through an ADV mechanism [51], thus being both micrometric and nanometric in size, depending on whether one considers the MB before or after ADV has taken place. For the treatment of heart disease, MBs are generally used to achieve ultrasound targeted microbubble destruction (UTMD) alone or in combination with an active principle such as miR-21, (GSK)-3β si-RNA, or FGF. In this way, they can favor angiogenesis [20], restore the presence of miR-21, which is essential for proper heart functioning [17], downregulate the expression of certain genes such as Gal-7 or (GSK)-3β genes to suppress a local immune response in the heart and hence allow heart grafting [17], or restore atherosclerotic plaque stability [52]. It has been suggested that the gene delivery method for the treatment of heart diseases such as myocardial infarction can be improved by combining UTMD with nuclear localization signal (NLS), which can facilitate DNA transfer from the cytoplasm to nucleus [53]. Through the activation of acidic fibroblast growth factor (FGF1 or aFGF-P), MBs can promote fibroblast development in cardiac tissue to prevent heart failure [18]. UTMD can also result in cavitation, hence promoting the migration of stem cells in areas where cell replacement is needed such as ischemic myocardium [16]. In addition to their applications in therapy, MBs can be used in diagnosis, for example, by providing an accurate measurement of intracardiac blood flow dynamics in the left ventricle through the so-called high-frame-rate echo-particle imaging using an optimal combination of MB infusion rate (1.2 mL/min) and mechanical indices (MI = 0.03–0.04), [54]. An additional interesting feature of MBs comes from their functionalization to enable their targeting of a part of the heart that is of interest such as the thrombus. The latter should indeed be detected and destroyed to avoid arterial thrombosis, for example, by using fucoidan as a targeting agent (TA) [13,55]. TAs, which are usually adsorbed on or covalently bound to MBs, can recognize, image, and destroy a receptor/molecule of interest (R) such as integrin, P-Selectin, or fibrin, which are known to lead to aggregation/interaction of platelets and the formation of thrombus, where examples of TA/R pairs consist of RGD/αIIbβ3, RGD/Glycoprotein IIb/IIIa, Fucoidan/P-selectin, tissue plasminogen activator (tPA)/fibrin, and CREKA peptide/fibrin [56]. Deng et al. [57] provide a detailed list of ligands that can be associated with NMs to target various cell receptors, where the nature of the ligand depends on the type of cardiovascular disease, which needs to be treated.

Overall, nanobubbles (NBs) are smaller than microbubbles (MBs), in other words, mostly below 1 µm, while MBs are generally larger than 1 µm. While NBs are made of a coating and internal compartment with a similar composition to MB (i.e., lipidic or polymeric external shell and gaseous core), the NB mode of action differs from that of MBs in that NBs can in principle more easily target heart compartments passively than MB due to their smaller sizes. Unlike MB, NB do not appear to act by a mechanism of bubble destruction similar to UTMD for the treatment of heart diseases. Instead, NBs can be associated with FGFP1 localized on their surface, resulting in the downregulation of CTGF, Casp-3 mRNA [29], and enclose a gas such as Xenon, which improves ultrasound imaging and reduces the volume of cerebral infarction by protecting oxygen/glucose deprived cells [23]; be linked with anti-CD3 or anti-CD4 antibodies to target T-lymphocytes issued from acute rejection (AR), hence highlighting the presence of AR, [21,24]; or be combined with anti-VEGFR-2 to detect atherosclerotic plaques [19].

Nanodroplets (NDs) are nanometric structures typically consisting of an external layer (e.g., polymeric PLGA-COOH), and an internal core (e.g., liquid dichloromethane (CH_2_Cl_2_),) which can transform into MBs under the application of ultrasound through a mechanism called acoustic droplet vaporization (ADV). As for NBs and MBs, NDs can be associated with molecules of interest for the treatment of heart diseases such as primary cardiomyocytes (PCMs) or 17β-estradiol (E2), which are drugs recommended for cardiac hypertrophy treatment [42]. When such NDs were injected intravenously into rats followed by the application of ultrasound in the heart region, it yielded both the release of E2 from NDs, and ND imaging following ND expansion into MB, highlighting a mechanism of ND use as a theragnostic compound for the treatment/detection of cardiac disease [42]. Other NDs encapsulating PFP liquid, which were labelled with a CNA35 targeting myocardial scar, could passively diffuse toward the fibrotic myocardium due to their small size, and transform into gaseous MBs under ultrasound application, enabling myocardial infarction detection [42].

Biodegradable polymeric nano-capsules (NCs) encapsulating a natural active principle (lychnopholide) recommended for the treatment of parasitic diseases were used to protect the organism against a sustained/continuous exposure of the organism to lychnopholides, and hence to prevent the well-known cardiac toxicity of lychnopholides [58]. Thus, NCs could potentially reduce side effects induced on the cardiac system by a significant number of drugs [59].

Hollow nanometric silica structures (HNSS) were designed with a size of 385 nm and an internal compartment content made of perfluorinated compounds to yield an ultrasound contrasting effect. Furthermore, they were associated with an antibody targeting hs-cTnI, which is a well-known marker of myocardial damage, thus allowing the detection by ultrasound imaging of acute myocardial infarction (AMI) following intravenous injection of HNSS to rats [33]. HNSS are characterized by two additional advantages. On the one hand, their stability can be achieved by an original method relying on the opposite polarities between the internal and external compartments of such structures. On the other hand, the presence of mesopores within silica promotes the attachment of compounds such as targeting agents to HNSS [33].

Finally, it is possible to take advantage of the properties of certain magnetic nanoparticles such as those composed of iron oxide, which can be grown inside the pores of silica meso-structures, hence increasing the stability of such structures and enabling them to act as bi-modal contrast agents (i.e., for ultrasound through the meso-structures that are suitable in sizes to yield ultrasound contrast and for MRI due to the well-known enhanced contrast provided by SPION) [34]. In addition, such combined structures can allow magnetic manipulation. When they are associated with an active compound such as an insulin-like growth factor, they can favor the release of such compounds from the meso-structures under controlled/sustained conditions (e.g., following ultrasound application), further yielding beneficial therapeutic effects characterized by an increase in stem cell viability, resulting in an enhanced efficacy of stem cell therapy [60].

## 3. Nanoscale Ultrasound Contrast Agents for Targeting Specific Diseased Heart Regions

**Figure 2:**
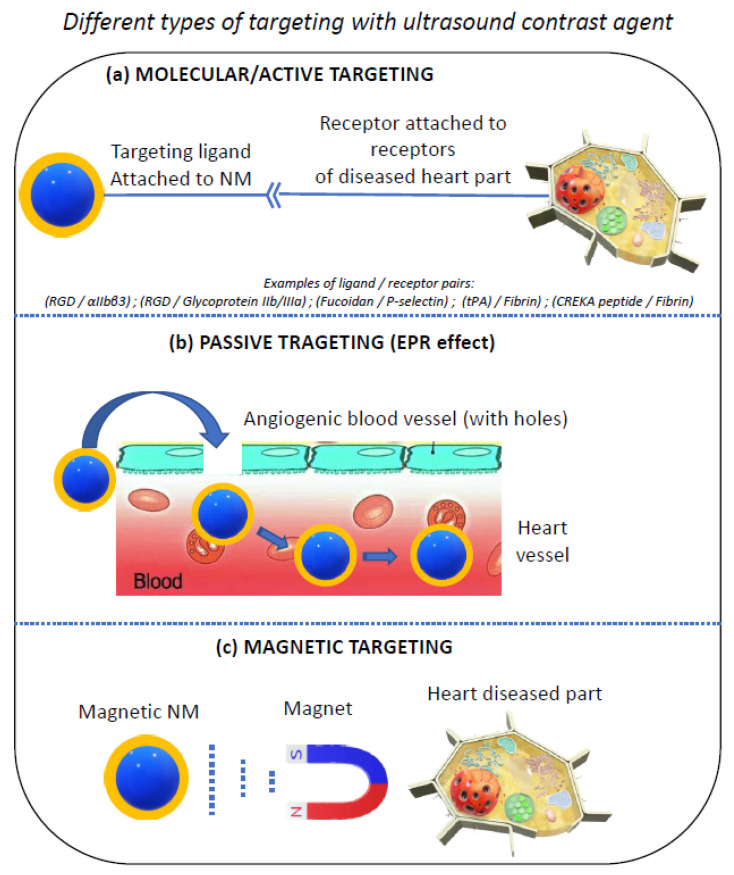
An illustration of the different ways in which a nanoscale contrast agent can target the DHR by relying on: (**a**) molecular/active targeting (i.e., a ligand is attached to the NM that specifically recognizes a receptor of the DHR), (**b**) passive targeting (i.e., NMs diffuse through the holes of the blood vessels irrigating the DHR), (**c**) magnetic targeting (i.e., a magnetic field is applied in the direction of the DHR, which attracts the magnetic NM toward the DHR).

Nanoscale contrast agents offer the advantage of being able to target regions of the diseased heart, essentially through three methods: passive, active, and magnetic targeting, as illustrated in Figure 2. Certain regions of the diseased heart such as those containing atherosclerotic plaques display open pores with sizes typically ranging from 380 to 780 nm. First, it has been shown that NBs can extravasate through these pores, thus allowing for visualization of the micro-vascularization of atherosclerosis plaques [19,61,62]. Second, certain ultrasound contrast agents such as MBs could also target a specific site of interest by being associated with a compound that specifically targets such sites, further enabling imaging. For example, MBs have been linked to maleimide, which targets selectins [63], cell adhesion molecules that are responsible for immune cell recruitment following ischemic injury [64]. Such MB complexes have enabled the detection of mouse heart inflammation, [63]. Another way of carrying out the targeting consists of using MBs to protect an active principle, so that the latter remains inactive in the absence of US application and becomes activated when it is released from the MBs by applying ultrasound at the site of interest. For example, MBs containing RNA therapeutics encapsulated within their external shell were injected intravenously to mice, leading to MB destruction and cavitation following US application in the heart, further resulting in the delivery of antimiR-23a to cardiomyocytes and the decrease in cardiomyocyte hypertrophy [65]. MB targeting can be used not only to enhance the imaging resolution of the heart site of interest, but also to trigger a therapeutic effect. For example, MBs conjugated, on one hand, with single-chain anti-GPIIb/IIIa antibodies (scFvs) that target specific thrombi receptors, and on the other hand, with scuPA, which displays a fibrinolytic activity, were able to image and destroy thrombi thanks to a combined targeting/pharmaceutical effect [66]. Furthermore, MBs captured by macrophages, which leads to a reduction in MB circulation time and results from the binding of serum proteins to MB, can be prevented by associating MBs with PEG molecules [67]. Third, magnetic targeting can be achieved to target specific parts of a diseased heart, for example, MBs of less than 7 µm associated with smaller 5–7 nm Fe_3_O_4_ NPs were used to target myocardial infarction in rats under magnetic field application, further helping these rats to recover their normal cardiac function [68].

## 4. Improved Ultrasound Imaging with Nanoscale Contrast Agents

**Figure 3:**
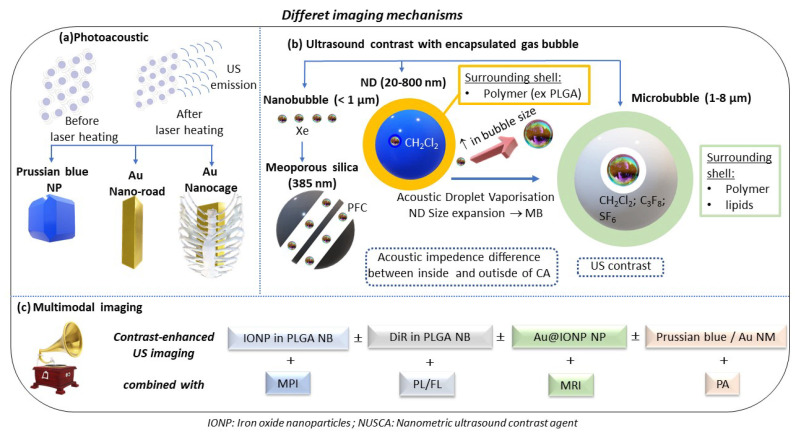
A schematic presenting the different mechanisms for producing ultrasound contrast (i.e., through the emission of ultrasounds resulting from the thermal expansion of a metallic NM heated by a laser (photoacoustic imaging) or via the difference in acoustic impendence between the gaseous inner part and the solid surrounding tissue of NM such as microbubbles, nanobubbles, or hollow silica meso-structures).

Different contrast agents can be used to enable local imaging using ultrasound, as presented for photo-acoustic and ultrasonography in Figure 3 by schematically summarizing the methods and associated materials used to generate contrasts in both cases.

The benefit of nanomaterials in detecting certain damaged or abnormal parts of the heart was highlighted by imaging carotid intima-media thickness (IMT) and plaques, which can preclude cerebrovascular events using either B-mode ultrasound or contrast enhanced ultrasound with MBs. In over 175 patients, it appeared that IMT and plaques were better visualized with contrast enhanced ultrasound (CEUS) than with B-mode ultrasound, suggesting that CEUS can be more efficient in detecting these heart abnormalities than the conventional B-mode [69]. In echocardiography, MBs can produce a local contrast, which comes from the difference in ultrasound reflection between the MB region and its surrounding, and can further enhance the resolution of the detection of heart abnormalities. To maintain the contrast, MBs should not be destroyed during ultrasound application, a situation typically reached for MI < 0.4 (i.e., when the pressure applied by the ultrasound on MBs is moderate enough) [70]. In addition, one should accurately choose the ultrasound frequency that determines the strength of the coupling between the US wave and the MBs, and has an impact on the contrast resulting from such interactions. By increasing the US frequency, the US wavelength decreases to approach a value close to the MB size, hence improving the resolution of the contrast. However, when the ultrasound reaches a high frequency, the penetration depth decreases, and ultrasound imaging cannot be carried out so deeply in the tissue [71]. 

For MBs used or occurring in two different ways (i.e., either directly injected intravenously to patients [72] or resulting from a heart disease condition such as intrapulmonary vascular dilations of microbubbles [73]), the ultrasound frequencies used to observe such MBs in two different heart chambers (i.e., the left atrium and the right ventricle) were between 2.5 and 3.5 MHz, hence representing typical ultrasound frequencies used for imaging MBs in the heart region [72,73]. 

A further refinement of US imaging in the presence of MBs consists in detecting US frequencies emitted by MBs, which are different from those of incident US due to US scattering by MBs. This method, designated as harmonic or sub-harmonic US imaging, can distinguish ultrasounds that have encountered MBs from those that have travelled through tissues. It relies on the behavior of US frequencies, which remain unchanged in tissues, and change following US interaction with MBs [74]. In addition, this technique can be carried out at high US frequency, further enhancing the US imaging resolution, thus yielding successful detection of micro-vessels in the mouse heart [74].

A commonly employed method to increase US resolution relies in using high frequency US imaging (HFUS) for ultrasound frequencies between 15 and 30 MHz [75], and ultra-high frequency US imaging (UHFUS) for ultrasound frequencies above 30 MHz [76]. An US of 12–15 MHz yields a typical resolution of 200 µm [77], which is insufficient to visualize heart microstructure tissues. UHFUS enabled typical resolutions of 67 µm to be reached at 40 MHz [78] and 30–92 µm at 70 MHz [79]. Such high resolutions have enabled the visualization of zebra fish heart [78], or the examination of zebrafish cardiac functions during heart regeneration [79]. However, HFUS and UHFUS are prone to a series of disadvantages. First, the US penetration depth, which is inversely proportional to the US frequency is limited in HFUS/UHFUS. Second, HFUS/UHFUS requires the design of specific high frequency transducers with miniaturized transducer elements that can be difficult to fabricate. Third, conventional MBs are too large to work at high frequencies (>15 MHz), and smaller MBs such as those of ~1 µm containing a C_4_F_10_ gas core surrounded by DSPC-DPPC shell [80] should be designed for HFUS/UHFUS.

In addition to HFUS/UHFUS, ultrasound super-resolution (USR) has been introduced to improve the resolution of traditional ultrasound imaging techniques whose spatial resolution is insufficient to visualize heart regions of interest such as the angiogenic vasa vasorum, which can highlight atherosclerotic plaque progression [20,81]. USR consists of ultra-rapid detection of an US signal scattered by MBs, further enabling the localization of these MBs beyond the acoustic diffraction limit with a subwavelength resolution (i.e., λ/5 for an US frequency of 7.7 MHz). It yields a spatial resolution enabling the visualization of tiny vessels with a 41 μm diameter [81].

## 5. Photoacoustic Imaging to Detect Heart Abnormalities

In addition to the methods described above that solely use US for excitation and detection, it is possible to rely on imaging methods that combine US detection with another type of radiation such as a laser to trigger the excitation of a nanometric US contrast agent (CA) [82,83]. Replacing the US excitation source with a laser to carry out so-called photoacoustic (PA) imaging enables the type of nanoscale ultrasound contrast agent (NUSCA) to be extended to materials other than MBs. In PA, the CA undergoes thermoelastic expansion following laser excitation, resulting in the emission of an ultrasonic wave that is detected by an US transducer that reconstructs the PA image. Some of the PA CA are endogenous chromophores such as melanin, [84] oxyhemoglobin/deoxyhemoglobin [85], lipids [86], or collagen [87]. Endogenous CAs do not display a specific localization in the part of the heart with abnormalities that need to be imaged. For this reason, exogenous PA CAs have been introduced whose properties should be optimized to enable an efficient coupling between the incident laser light and nanomaterials (NM). These CAs should display a low quantum yield to prevent the light absorbed by the laser from being converted into emitted photons, a high molar-extinction coefficient to allow optimal absorption of laser energy by PA CAs, and an absorption in the near-infrared (NIR) at which laser light can penetrate sufficiently deep inside the tissues to be imaged without being absorbed by the organism. These properties are often brought together in NMs with so-called localized surface plasmon resonance (LSPR). Some metallic NMs, especially Au NPs, fulfil such properties. In addition to being plasmonic, Au NMs can be produced with various geometries and aspect ratio (e.g., nanorods [88], nanospheres [89], and nanodisks [90], enabling, on one hand, the optimization of their absorption properties and associated plasmonic behavior, and on the other hand, to tune their surface/volume ratio and hence to adjust their biodistribution properties and the number/type of molecules attached to them such as PEG or targeting ligands [91]. Furthermore, Au NMs can be combined with other metallic structures to yield mixed metallic NMs such as PEGylated core-shell Pd@Au nanoplates [92]. Other metallic NMs such as Ag nanocrystals [93] and Pd nanosheets [94] have been introduced as potential PA CAs. Non-metallic NMs, with intrinsic plasmonic properties or plasmonic behavior reached through their association with plasmonic materials, have also been suggested such as TiS_2_ [95] or various carbon-based NMs such as carbon nanotubes [96]. The combination of CAs and PA has been used advantageously for the treatment and detection of heart diseases in the following manner. First, Prussian blue nanoparticles (PBNPs) coated with PLL were internalized in stem-cells, hence enabling the optoacoustic imaging of these cells down to a resolution of 200 cells/μL in vivo [97]. Second, NPs can be used to deliver drugs for the treatment of cardiac disease such as resveratrol to mesenchymal stem cells (MSCs) and then to visualize MSCs by PA [98]. Third, coupling of NMs with cell penetrating peptides (CPPs) to tag human embryonic stem cell-derived cardiomyocytes (hESC-CM) and image by PA these cells with a resolution down to 2000 cells [99]. Fourth, improved targeting can also be achieved by a coating such as silica, which prevents NP aggregation, leading to silica coated Au nanorods yielding more efficient MSC targeting and imaging than uncoated Au nanorods [88]. Fifth, nanomaterials such as citrate coated Prussian blue particles have been imaged with PA to monitor dynamic phenomena such as the rehabilitation over time of damaged vasculature [100]. Sixth, in order to increase the depth of penetration of laser radiation, PA imaging can be implemented using two-photon or three-photon PA imaging, enabling, for example, the monitoring of MSCs labeled with gold nanocages [101]. Seventh, PA can be combined with MRI by using NMs such as IONPs surrounded by a Au coating acting as contrast agents for these two imaging modalities [99], a dual imaging method used to confirm the delivery of MSCs into the brain. Eighth, PA can be used in conjunction with US [99], using plasmonic gold nanospheres to label MSCs, where ultrasound provides images of the patient’s surface vasculature and PA identifies the variation of relevant physiological parameters such as oxygenated and deoxygenated blood. Ninth, three modal imaging methods can even be carried out (e.g., by combining PA with magnetic particle imaging (MPI) and US) for tracking cardiac stem cells [22] by using PLGA NB enclosing IONP and coated with DiR. In this case, the different imaging modalities present specific and complementary advantages (i.e., MPI enabled by the presence of IONP favors deep tissue imaging, PA generated by DiR yields enhanced contrast in soft tissue structures, and US provided by the NB displayed a high temporal resolution).

## 6. Therapeutic Activities of Nanoscale Contrast Agents Exposed to Ultrasound against Heart Diseases

**Figure 4:**
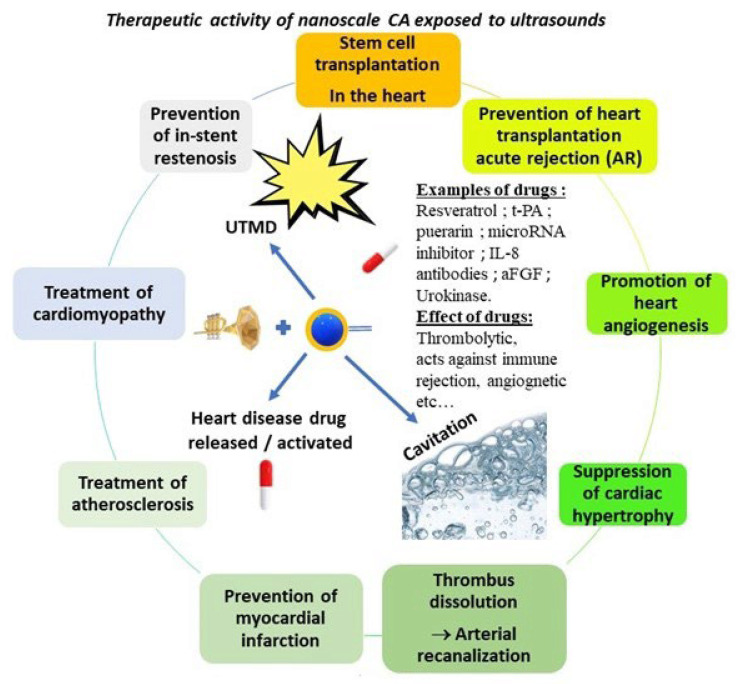
A schematic summarizing the various mechanisms under which an ultrasound contrast agent can trigger a therapeutic activity (i.e., through cavitation, ultrasound targeted microbubble destruction, or the release/activation of a heart disease drug). The different heart conditions that can be treated with these methods are listed (i.e., the suppression of cardiac hypertrophy, the destruction/dissolution of thrombi leading to arteria recanalization, stem cell transplantation in the heart, prevention of heart transplantation acute rejection, promotion of heart angiogenesis, prevention/treatment of myocardial infarction, and treatment of atherosclerosis).

The various therapeutic activities of nanoscale contrast agents exposed to ultrasounds are summarized in Figure 4.

Sonothrombolysis, which is carried out by treating thrombi with a combination of ultrasound and nanoscale ultrasound contrast agents (CA), presents a number of advantages compared to the use of ultrasound alone. First, CA can help transport thrombolytic agents such as t-PA or urokinase to the blood clot [102]. Second, thrombus destruction can be enhanced when NSCA penetrates the thrombus, a situation that can be met by using CAs of small sizes (<100 nm) or CA acting as a cavitation nuclei, which enhances the cavitation effect of ultrasound at clot location, hence favoring clot destruction/dissolution [103]. Third, diagnostic ultrasound parameters such as ultrasound frequencies of 2–3 MHz can be used in sonothrombolysis, making this method implementable with standard US apparatus. Fourth, when a thrombolytic agent such as t-PA is encapsulated in MBs, it is protected against degradation and can therefore be injected at a lower dose than in the absence of MBs, hence reducing the potential side effects of this agent such as hemorrhage [104]. Fourth, the thrombolytic agent (THA) can be encapsulated in MBs together with a contrasting gas (perfluorocarbon), hence enabling thrombi to be both detected and destroyed with MBs [105]. Fifth, the THA can be delivered from MBs under controlled conditions of ultrasound application, resulting in encapsulated t-PA having a longer half-life than non-encapsulated t-PA [106]. Sixth, targeted microbubbles can be used that directly target clot ligands such as fibrin, hence improving arterial recanalization, which is one of the main desired outcomes of thrombi destruction [107]. Seventh, by using magnetic microbubbles, it is possible to increase MB blood clot targeting with the help of a low strength magnet (0.08–0.38 T), which attracts MBs in the clot region, hence resulting in enhanced blood clot lysing rates under ultrasound application [108].

Sonodynamic therapy (SDT) is a method, which originates from photodynamic therapy (PDT) [109], but uses ultrasound instead of light to activate ultrasound activable compounds, designated as sonosensitizers. SDT presents the advantages of being compatible with the use of a large number of photosensitizers, hence resulting in a wide choice of potential sonosensitizers and of enabling deeper tissue penetration than PDT. SDT can benefit from the use of nanomaterials for the treatment of heart diseases (e.g., Au NPs can be loaded with the sonosensitizer to treat atherosclerosis by SDT [110], or MBs containing puerarin and sulfur hexafluoride can be used to improve delivery and pharmaceutical efficacy of puerarin in the treatment of diabetic cardiomyopathy (DCM)) [111]. Sonodynamic therapy (SDT), which consists of activating a so-called sonosensitizer under the application of low-intensity ultrasound, can treat both atherosclerosis through the prevention of atheromatous plaque formation or the increase in plaque stability [110] and diabetic cardiomyopathy (DCM) (e.g., by improving the activity of a DBM drug such as puerarin [111]). By using a NM as a sonosensitizer, US contrast can be improved, for example, by using MBs, targeting of the DHR can be achieved through active, passive, and/or magnetic targeting depending on NM type, and on-demand/on-site activation could be realized by associating a DBM drug to a NM, which is activated/released by the application of low intensity ultrasound.

## 7. The Various Heart Diseases that Can Be Detected/Treated By Using a Combination of Nanoscale Contrast Agents and Ultrasound

It therefore appears that NMs used in combination with ultrasound can improve the detection and treatment of many of the previously mentioned heart diseases. For example, mesenchymal stem cells (MSC), which are primarily found in the bone marrow and therefore also designed by bone marrow mesenchymal stem cells (BMSC), can be transplanted to damaged parts of the heart (myocardial infarction) to favor the regeneration and repair of such regions [112]. Thus, a treatment of rats suffering from acute myocardial infarction (AMI) treated with BMSC transplantation in the presence of ultrasound targeted microbubble destruction decreased galectin-7 expression or SDF-1/CXRC4 upregulation and thus promoted such transplantation and further resulted in improved cardiac functions [113,114].

Concerning human embryonic stem cell cardiomyocytes (hESCC), they can potentially treat heart failure by enabling heart regeneration. To make this approach successful, hESCC cell transplantation should be monitored by using, for example, polymeric nanoparticles acting as PA contrast agents that can be used to follow hESCC-CM transplantation in living mouse hearts [100].

The success of a stem cell-based heart disease treatment relies in large part on the use of an efficient imaging method to monitor stem cell transplantation. Thanks to nanomaterials, this can be achieved either by using contrast enhanced echography with NMs [115], or PA imaging in the presence of specific NMs such as certain types of metalloporphyrins (i.e., cobalt protoporphyrin IX (CoPP) [116], where CoPP introduced in mesopores can yield an enhanced signal compared with free CoPP and be released in a sustained manner to improve the quality of in vivo imaging).

Furthermore, angiogenesis can occur or be triggered during/following a heart disease for the benefit of the patient (i.e., when the blood flow toward the heart decreases, new angiogenetic vessels can grow to overcome a ischemic insult [68]). For example, acidic fibroblast growth factors (aFGF), which are known to promote angiogenesis as shown when released from encapsulated polycaprolactone (PCL) [113], are associated with cationic lipid microbubbles, resulting under UTMD in improved heart function in rats [18]. MBs exposed to US were used to treat rats suffering from diabetic cardiomyopathy (DCM), resulting in improved heart vessel vascularization and in increased cardiac perfusion [65]. Another approach consisted of encapsulating VEGF in polymeric NP to increase heart vascular density, reduce the size of infarcts, and overcome heart dysfunction [117].

Heart transplant, which is carried out when patients have reached the latest stage of heart failure/disease, can also benefit from a treatment involving MBs exposed to ultrasound, as shown by detecting the presence of acute cardiac transplant rejection (AR) with MB targeting intercellular adhesion molecule-1 (ICAM-1) [118], or T lymphocytes [21], which both increase in number following AR. The detection of such MBs in the targeted region by US imaging could hence reveal the presence of AR.

Hypertrophic cardiomyopathy (HCM) is characterized by an increase in heart muscle thickness, resulting in the heart acting as a less efficient pump. It was shown that MBs targeting the microRNA inhibitor in the presence of UTMB could suppress cardiac hypertrophy in mice [65].

Atherosclerosis, which can cause heart failure, is characterized by the deposition of a lipid plaque on the walls of the arteries. UTM can be used to deliver IL-8 antibodies, reduce the inflammatory response, and increase plaque stability in a rabbit atherosclerosis model [52]. MBs can be associated with a nanobody targeting vascular cellular adhesion molecule 1 (VCAM-1) to target atherosclerosis plaques that induce VCAM-1 and image them [119]. A mouse model of atherosclerosis injected with MB associated with biotinylated antibody targeting ICAM1 and the angiogenesis inhibitor Endostar (MBie) inhibited atherosclerotic plaque in a mouse model of atherosclerosis in the presence of UTMB [120].

A thrombus, also called a blood clot, results from blood coagulation in heart vessels. It can decrease the amount of blood flowing from or toward the heart. MBs combined with the application of ultrasound can be used to dissolve/destroy thrombi through the delivery of thrombolytic drugs, mechanical stress induced by acoustic cavitation, or UTMD occurring/performed in the region of the thrombi either by applying ultrasound in this region or by using MB ligands such as RGDS tetrapeptide that target thrombi. These methods were able to achieve complete recanalization of the blocked artery, under possible local monitoring of thrombus destruction using highly contrasting MBs [121].

Cardiomyopathy, which is characterized by the presence of a dysfunctional heart muscle preventing the heart from properly pumping blood to other parts of the body, is described as occurring in patients suffering from diabetes or treated with certain drugs such as doxorubicin, [11,26,29,36,37,46,111]. Combining nanometric contrast agents (MBs) with a targeting ligand (FGP1) makes it possible to bring FGP1 specifically in the DHR, where FGP1 can promote endothelial vascular tissue/smooth muscle cell proliferation. In addition, FGP1 can be released in a controlled manner by the application of the ultrasound, leading to therapeutic activity localized in the DHR, which can even be enhanced by triggering UTMD in this region [11,26,29,36,37,46,111].

In-stent restenosis is characterized by the narrowing of a blood vessel following stent implantation, leading to the slow-down or blockage of blood flow and to heart attack in the worst-case scenario. To prevent such adverse events from occurring, vascular polymeric nano-patches embedding anti-restenotic drugs can be used to release such active principles locally in the DHR under ultrasound application [28].

## 8. Conclusions

**Figure 5:**
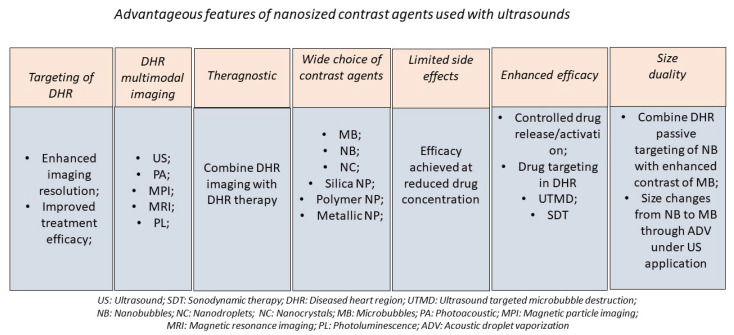
A list of advantageous features of nanosized contrast agents used with ultrasounds comprising: (i) the targeting of the diseased heart region resulting in the imaging/treatment of this region; (ii) the possibility to carry out multimodal imaging (US; PA; MPI; MRI; PL) and hence to benefit from the combined advantages of these different imaging methods; (iii) theragnostic properties (i.e., imaging and therapy can be combined); (iv) a wide choice of contrast agents is available including MBs, NBs, NDs, NCs, Silica NMs; Au NMs; (v) the efficacy of the treatment against heart diseases can be achieved at reduced drug concentration, hence minimizing the potential side effects of such drugs; (vi) the heart disease drug can be released/activated under the controlled condition by deciding to apply the ultrasound or not and by applying the ultrasound in the diseased heart region; (vi) the size of some NMs can be tuned (i.e., NDs can be transformed into MBs under acoustic droplet vaporization), hence enabling such system to benefit from the enhanced targeting efficacy of NDs (i.e., the EPR (enhanced permeability and retention) effect was improved for NDs of relatively small sizes and MBs yielded superior echogenicity).

Cardiovascular diseases (CVD) were responsible for the death of 18 million people in 2019, representing the cause of one third of all global deaths [122], hence necessitating the development of new diagnosis/treatment methods of CVD to reduce this number. Among such methods, ultrasound presents the advantage of being safe, painless, non-invasive, relatively inexpensive, and of enabling the imaging of internal heart structures. The combination of ultrasound with nanomaterials brings a series of additional appealing features, as summarized in Figure 5. First, the contrast can be generated specifically in the DHR by using NMs that target such regions through passive, active, or magnetic targeting (i.e., the resolution of the imaging can be improved by enhancing the ultrasound contrast in the DHR. Second, while NMs filled with a gas are used in echography to improve the contrast through a difference in acoustic impedance between the gaseous content of the NM inner part and the non-gaseous NM surrounding made of heart tissue, metallic NMs, which display a so-called surface plasmonic effects under laser irradiation, can be employed in photo-acoustic imaging, hence resulting in a large choice of NM potentially usable for US imaging. Furthermore, by skillfully adjusting NM composition, it is possible to combine US imaging with other imaging modalities such as magnetic resonance imaging, optical imaging, and magnetic particle imaging. Third, the presence of nano-scale contrast agents makes a therapeutic approach feasible by relying on the controlled release and activation under ultrasound application of a heart disease drug associated with a NM, a mechanism that can be further enhanced in the presence of ultrasound targeted MB destruction or cavitation. Among the various heart diseases that have been described as being treatable by this combined NM/US approach are atherosclerosis, [26], heart attacks, ischemic heart disease, [123], myocardium infarcts, [124], thrombosis, and cardiac hypertrophy, [125] where the presence of NMs improves the efficacy of sonothrombolysis, triggers the activation of a specific heart disease drug or of a sonosensitizer via sonodynamic therapy, or prevents acute rejection following stem cell transplantation in the DHR.

NM biosafety, which is a prerequisite for NM human administration, depends on several interdependent factors such as NM administration route, physico-chemical properties or compositions [126]. Therefore, it is difficult to draw general conclusions about NM biosafety. The latter needs to be assessed on a case-by-case basis. For certain types of NMs such as silica NPs [126], liposomes [127], polymer NPs [128], or iron oxide NPs [129], conditions that could guarantee their biosafety have been suggested in terms of specific values of NM concentration, administration route, formulation, size, charge, and/or composition.

## Figures and Tables

**Table 1:** Properties of various ultrasound contrast agents used for imaging and therapeutic applications including size of these materials, origin of echogenicity, imaging results, combination with therapy, and applications.

Nanosystem	Size (nm)	Origin of Echogenicity	US Parameters	Targeting/Activity	Imaging Results	Therapeutic Results	Applications	Ref
**MICROBUBBLES (MB)**								
nanopackaged tissue-type plasminogen activator (t-PA) gene plasmid cross-linked to ultrasonic MB composed of sucrose and bovine serum albumin	2000 to 5000	MB inner gas	Therapeutic ultrasound: f = 1 MHz; i = 1.5 W/cm^2^; t = 10 minutes;	t-PA: serine protease cleaving plasminogen into active plasmin for fibrin digestion in thrombus;	Following iv injection of MB/t-PA: Before therapeutic ultrasound applied: MB/t-PA observed by US in heart; After therapeutic ultrasound applied: MB/t-PA disappear from US imaging in heart;	Treatment steps: i) valve replacement performed; ii) injection of MB/t-PA; iii) application of therapeutic ultrasound on the heart; iv) high expression of t-PA in myocardium; v) Prevention of thrombosis 2 months after valve replacement	Avoiding thrombosis after mechanical heart valve replacement	[10]
MaFGF-loaded NP (MaFGF-NP) + MB	132	MB inner gas	f = 12–14 MHz;	None	None	(MaFGF-NP) + MB destruction: ↓ left ventricular dysfunction, myocardial fibrosis, cardiomyocytes apoptosis and oxidative stress.	Prevention of DOX-induced cardiomopathy	[11]
PTA-PTX NP encapsulated in shell of magnetic microbubble (MMB-PLGA- PTX)	6000	MB inner gas	f = 10–900 kHz; t = 4 sec.;	Drug targeting stent under magnetic field application; Drug released from stent under US application;	none	MMB-PLGA-PTX: magnetic targeting of stent; low intensity focused ultrasound (LIFU): production of stable microbubble oscillations → release of PLGA-PTX;	in-stent restenosis treatment	[12]
Polymer (PIBC) MB conjugated with fucoidan (Fucoidan-MB)	2000 to 6000	Air inside MB	f = 40 MHz;	Fucoidan target target P-selectin in thrombus	Fucoidan-MB: localize/image rat thrombotic wall; Fucoidan-MB: Not present in healthy vein;	None	Imaging of thrombus	[13]
nano lipid MB (DPPC + DSPE-PEG) conjugated to anti-ICAM-1 (MB-anti-ICAM-1)	683	C_3_F_8_	f = 14 MHz	ICAM-1 (marker of atherosclerosis)	MB-anti-ICAM-1 located/imaged in vascular wall of abdominal aorta	None	identifyi inflammatory injury due to atherosclerosis	[14]
miRNA in exosomes + UTMD with MB (sonovue)	30–150	MB inner gas	f = 40 MHz (US imaging); f = 0.7 MHz t = 1 min (in target area for UTMD);	UTMD for targeted delivery of exosomes to the heart	none	Exosome + miRNA delivered in mouse heart using UTMD → restores cardiac function (following doxorubicin induced cardiotoxicity)	Protects the heart from chemotherapy related cardiotoxicity	[15]
MB+ bone marrow stem cell (BMSC) transduced with lentiviral PHD2 shRNA	1000	C_3_F_8_	f = 10 MHz (echography) f = 1 MHz; i = 2 W/cm2; (UTMD)	UTMD	Imaging of MB in heart	Transplantion of PHD2 shRNA-modified BMSC in presence of UTMD: ↓ infarct size, ↑ vascular density, and ↑ cardiac function; PHD2 silencing: ↑ BMSC survival through a HIF-1α-dependent mechanism;	Improve stem cell therapy following acute myocardial infarction.	[16]
Galectin-7-siRNA (siGal-7) bound to cationic MB (CMB)	424 (CMB)	C_3_F_8_	f = 1 MHz; t = 2 min; i = 2 W/cm^2^; (UTMD)	siRNA stops translation of Gal-7 (Gal-7: responsible for rejection of grafted heart);	none	galectin-7-siRNA-CMB + UTMD: stops acute cellular rejection following allograft heart transplantation	acute cellular rejection (AR) after heart transplantation (HT)	[17]
Complex of: aFGF–NP + cationic lipid microbubbles (CPMB)	4390	SF_6_ gas	f = 4 MHz; Pulsed mode; (UTMD)	Through UTMD in heart aFGF: targets myocardial tissue +	M-mode echocardiography of heart with Nano-complex + UTMD	aFGF–NP + CPMB + UTMD prevents left ventricular dysfunction due to DOX. aFGF ↑ vascular endothelial/smooth muscle cells proliferation → treatment of HF; NP + aFGF: ↑ half–life, stability, efficacy of free aFGF;	Treatment of Doxorubicin-Induced Heart failure	[18]
DSPE-PEG2000 NB conjugated with anti-VEGF-2 (NB-VEGF2)	320	SF_6_	NA	Active targeting: anti-VEGF-2: targeting angiogenesis (dominant in atherosclerosis) Passive targeting: EPR effect	US detection of rabbit abdominal aorta atherosclerotic plaquet using NB-VEGF2 as targeted contrast agent	None	Detection of atherosclerosis	[19]
**NANOBUBBLES (NB)**								
NB (Nanobubbles) + anti-CD25 antibody	420	Perfluoropropane	f = 7–14 MHz;	Anti-CD25 antibody targeting T cells in in myocardium	MCE + NB + antibody → imaging of acute rejection (AR) after heart transplantation	None	Detection of Acute rejection after heart transplantation	[20]
NB + anti-CD3 antibody (NB-CD3)	460	C_3_F_8_ inside NB	NA	NB-CD3 target T lymphocytes	US signal of NB-CD3 proportional to number of T lymphocytes	None	Detection of acute rejection detection after cardiac transplantation (through detection of T lymphocyte infiltration)	[21]
(PLGA)-IONP NB conjugated with (DiR)	185 (IONP: 4)	NA	f = 21 MHz	None	Trimodal imaging: US + PA + MPI; US: real time guidance; PA (DiR): ↑ contrast; MPI (IONP): deep tissue imaging NB injected in myocardium: Signal of stem cells in cardiac tissue increased by 4 (US), 10 (PA), 20 (MPI).	None	Stem cell therapy	[22]
NB encapsulating Xe (Xe-NB)	225	Xe gas	f = 18 MHz;	accumulation or aggregation of NB to ischemic lesion	Xe-NB in ischemic lesion: contrast due to accumulated Xe-NB;	Xe-NB therapeutic effects: protects oxygen/glucose-deprived PC12 cells against apoptosis; Restores vascular circulation in lesion area; Reduces volume of cerebral infarction; Restore neurological function;	Treatment of Acute Ischemic Stroke	[23]
NB conjugated with anti-CD4+ (NBCD4)	545	NA	f = 5.6 MHz; MI = 0.085;	NBCD4: Targeting of CD4+ activated by ACAR	US signal of NBCD4: ↑ in animals prone to ACAR due to infiltrating CD4+ lymphocytes; Detect the presence of ACAR	None	Acute cardiac allograft rejection (ACAR) after heart transplantation	[24]
**NANODROPLETS (ND)**								
Fe_3_O_4_ NP + PFH inside nano-capsule (DSPE-PEG2000-IMTP + DPPC + Cholesterol)	348	Gas (Perfluorohexane) Liquid vaporizes to gas phase under US activation (ADV);	f = 1 MHz; i = 1–4 W/cm^2^; t = 1–4 min; pulsed wave (low intensity ultrasound)	Targeting of ischemic region of myocardium: Passive targeting: EPR; Active targeting: Ischemic myocardium-targeted peptide (IMTP)	Trimodal imaging: US + PA + MRI; Imaging of ischemic myocardium in rat; Targeting of hypoxia-injured heart cells; Targeting of rat heart;	None	Targeting/imaging of ischemic/hypoxia injured heart cells	[25]
**POLYMER NP**								
Perfluorocarbon + SPIO in NP (polymer: PLA+PLGA-COOH+PFOB) + VEGFR-2 antibody	404	Gas (Perfluorocarbon)	MI=1.6; f = 15 MHz;	Endothelial VEGFR-2.	Bimodal imaging: US + MRI; Molecular imaging of atherosclerotic neovasculature; Detection of VEGFR-2+ endothelial cells, GSI, CNR, PPACD31+ and PPAVEGFR-2+;	None	prediction of plaque vulnerability	[26]
NP (PLA) encapsulating PFOB surrounded by OPN + Cy5.5	360	perfluorooctyl Bromide (PFOB)	f = 40 MHz (high frequency)	Osteopontin (OPN) to target VSMC	Bimodal imaging: ultrasound + optical Imaging of vascular smooth muscle cells (VSMC) VSMC involved in atherosclerotic plaque progession	None	Detection of Atherosclerotic Plaques	[27]
BaTiO_3_ NP + PLLA + PE + anti-restenotic drug sandwitched between PE/PLLA layers	100 (BaTiO3)	None	i = 20 W; f = 40 kHz; t = 10 sec;	Drug released from PE → antiproliferative effect on human smooth muscle cells yielding restenosis.	None	Anti-restenotic drug released from nano-patches under ultrasound application	Anti-restenotic treatment	[28]
PLGA NB bound to FGF21 (NB-FGF21)	880	C_3_F_8_ gas	f = 40 MHz; (imaging) f = 500 kHz; i = 2 W; t = 5 min; (LFUS)	LFUS enables controlled release of FGF21 FGF21: ↓ hypertensive cardiac remodelling, ↓ cardiac hypertrophy, ↓ inflammation ↓ oxidative stress caused by DOX.	Echocardiography: increase of contrast of cardiac chamber between before and after NB-FGF21 injection;	LFUS on NB-FGF21: accumulation of NB-FGF21 in myocardial tissue; downregulation of ANP, CTGF, and caspase-3 mRNA ↓ of myocardial hypertrophy, interstitial fibrosis in diabetic mice.	Treatment of diabetic cardiomyopathy	[29]
Fe_3_O_4_-poly(lactic-co-glycolic acid)-PFH-CREKA nanoparticles (NP)	311	PFH Perfluoro-hexane	i = 1 W/cm^2^;	CREKA peptide: targets fibrin of thrombus;	NP phase transition monitored by photoacoustic imaging; (Fe_3_O_4_ for MRI imaging)	NP phase transition (PT) under low-intensity focused ultrasound → vaporization of PFH to yield thrombolysis.	Treatment of thrombus	[30]
CNA35-PFP polymer NP (DPPC+DSPE+DSPG)	295	PFP Perfluoro-pentane (liquid−gas phase transition)	f = 5−9 MHz; i = 1−2 W/cm^2^; (Low Intensity Focused Ultrasound)	Passive targeting: CNA35-PFP NP diffuse through endothelial cell gap (EPR effect); Active targeting: CNA35-PFP NP target fibrosis in ischemic myocardium; → CNA35-PFP NP adhere to surface of fibroblasts in fibrotic myocardium;	iv injection of CNA35-PFP NP in animal model of myocardial infarction Followed by LIUF application: → Transform PFP from liquid to gaseous MB + ↑ US in fibrotic region;	None	Detection of Myocardial Fibrosis	[31]
Imatinib mesylate encapsulated in bioadsorbable polymeric NP	200	Contrast media: Iopamidol	f = 40 MHz;	Drug-eluting stents (DES) targeting vascular smooth cells	Ultrasound imaging to assess the extend of neointima formation	Imatinib-NP eluting stent: ↓ in-stent neointima + stenosis	Supression of neointima formation; Prevention of in-stent restenosis;	[32]
**SILICA NP**								
Silica nanosphere	338	perfluorodecyl Silane inside nanosphere	MI=1.3	Active targeting with anti-Cardiac Troponin I Antibody at surface of nanosphere	delineation of myocardial necrosis sites	None	Early Diagnosis of Acute Myocardial Infarction	[33]
Fe_3_O_4_ + IGF in pores of mesocellular foam silica NP	383 (16: pore)	mesocellular foam silica NP (MCS)	f = 40 MHz;	IGF: insulin-like growth factor → improve cell viability	Enhanced ultrasound signal in the presence of MCS	Release of IGF from nano-system: ↑ efficacy of stem cell therapy;	Stem cell therapy in heart disease	[34]
silica-based NP	300	Aggregated NP	F = 16–40 MHz;	None	NP: ↑ ultrasound contrast of labeled human mesenchymal stem cells (hMSCs); NP aggregation: ↑ US signal; ↑ resolution with US (down to 70 000 cells) than MRI (down to 250 000 cells)	NP: ↑ production of paracrine factors implicated in cardiac repair; NP: ↑ delivery of stem cells in the right location (avoid fibrotic tissue);	Stem cell therapy in heart disease	[35]
**NANOLIPOSOME**								
FGF1-loaded nanoliposomes (FGF1-nlip)	80	NA	f = 12 to 14 MHz	Acidic fibroblast growth factor (FGF1) → prevents diabetic cardiomyopathy.	Realtime myocardial contrast echocardiography: detection of left ventricular systolic function and perfusion changes in diabetic rats	FGF1-nlip + UTMD on diabetic rats: suppress cardiac abnormalities	Treatment of diabetic cardiomyopathy	[36]
Non-mitogenic acidic fibroblast growth factor (NM-aFGF) in PEGylated nanoliposomes (NM-aFGF-PEG-lips)	125	None	f = 12–14 MHz; MI = 1.9; T = 10 sec; (MB destruction)	combination of NM-aFGFPEG- lips and UTMD could achieve cardiac-targeted delivery	None	NM-aFGF-PEG-lips + ultrasound-targeted microbubble destruction (UTMD): improve cardiomyocyte structural abnormalities in animals with diabete	Treat cardiac abnormailities due to diabete	[37]
**OTHER TYPES OF NANOMATERIALS**								
hydrogen peroxide (H_2_O_2_)/perfluoropentane (PFP) phase-change NP	457	Perfluoropentane	Therapeutic ultrasound: f = 1 MHz; i = 1–8 W;	None	US applied on H_2_O_2_/PFP NP → Acoustic signal increases	Oxygen release under ultrasound application: ↓ myocardial reperfusion Injury	Treatmment of coronary thrombolysis	[38]
NP with t-PA + gelatin + zinc ions	100	None	transthoracic US f = 1 MHz; i = 1 W/cm^2^;	t-PA for thrombus destruction	None	US application: i-PA release from NP + t-PA activation in affected coronary artery → recanalization of occluded coronary	intracoronary thrombolysis	[39]
Basic fibroblast growth factor (bFGF) + NP (NP-bFGF)	128	MB	f = 14 MHz; MI = 1.9; t = 10 sec; (MB destruction)	Destruction of MB + NP in heart by ultrasound application	MB imaged to control MB destruction.	NP-bFGF + UTMD → deliver bFGF to the heart to trigger growth factor therapy → restore cardiac functions + damaged cardiac tissues.	Treatment of Diabetic cardiomyopathy	[40]
albumin-bound particle form of paclitaxel (nab-PTX)	130	None	NA	PTX to reduce restenosis	Ultrasound used for placing stent in coronary lesion	injection of nab-PTX after stent positioning → target lesion revascularizations (TLR)	reducing in-stent restenosis	[41]
Nano-probe encapsulating PFP and E2 conjugated with PCM;	418 nm	PFP (Perfluoro-carbon)	f = 5–12 MHz; i = 3.2 W/cm2 t = 10 min (low-intensity focused ultrasound imaging/therapy)	Targeting: primary cardiomyocyte (PCM) targeting primary cardiomyocyte; Activity: 17β-estradiol (E2) as anti-hypertrophic drug;	PCM-E2/PFP: imaging contrast agent.	PCM-E2/PFP + LIFUS: ↑ release of E2, ↓ systemic side effects; ↑ cardiac targeting (enhanced drug circulation time); ↓ cardiac hypertrophy;	Treatment of cardiac hypertrophy	[42]
Acoustically-responsive fibrin scaffold (ARS) containing basic fibroblast growth factor (bFGF)	14,000	PFH	F = 2.5 MHz; Pressure = 2 Mpa;	bFGF: pro-angiogenic growth factor to stimulate blood vessel formation and restore perfusion;	Imaging of gas bubbles generated by ADV (pressure > 2 Mpa) in ARS.	Release from ARS of bFGF under ultrasound application: perfusion and blood vessel density;	treatment of vascular disease	[43]
